# Expression of class II histone deacetylases in two mouse models of temporal lobe epilepsy

**DOI:** 10.1111/jnc.13440

**Published:** 2015-12-28

**Authors:** Rohan Jagirdar, Meinrad Drexel, Anneliese Bukovac, Ramon O. Tasan, Günther Sperk

**Affiliations:** ^1^ Department of PharmacologyMedical University InnsbruckInnsbruckAustria

**Keywords:** epileptogenesis, granule cell dispersion, HDAC9, histone deacetylases, status epilepticus, temporal lobe epilepsy

## Abstract

Epigenetic mechanisms like altered histone acetylation may have a crucial role in epileptogenesis. In two mouse models of temporal lobe epilepsy, we investigated changes in the expression of class II histone deacetylases (HDAC), a group of signal transducers that shuttle between nucleus and cytoplasm. Intrahippocampal injection of kainic acid (KA) induced a status epilepticus, development of spontaneous seizures (after 3 days), and finally chronic epilepsy and granule cell dispersion. Expression of class II HDAC mRNAs was investigated at different time intervals after KA injection in the granule cell layers and in sectors CA1 and CA3 contralateral to the site of KA injection lacking neurodegeneration. Increased expression of HDAC5 and 9 mRNAs coincided with pronounced granule cell dispersion in the KA‐injected hippocampus at late intervals (14–28 days after KA) and equally affected both HDAC9 splice variants. In contrast, in the pilocarpine model (showing no granule cell dispersion), we observed decreases in the expression of HDAC5 and 9 at the same time intervals. Beyond this, striking similarities between both temporal lobe epilepsy models such as fast decreases in HDAC7 and 10 mRNAs during the acute status epilepticus were observed, notably also in the contralateral hippocampus not affected by neurodegeneration. The particular patterns of HDAC mRNA expression suggest a role in epileptogenesis and granule cell dispersion. Reduced expression of HDACs may result in increased expression of pro‐ and anticonvulsive proteins. On the other hand, export of HDACs from the nucleus into the cytoplasm could allow for deacetylation of cytoplasmatic proteins involved in axonal and dendritic remodeling, like granule cell dispersion.

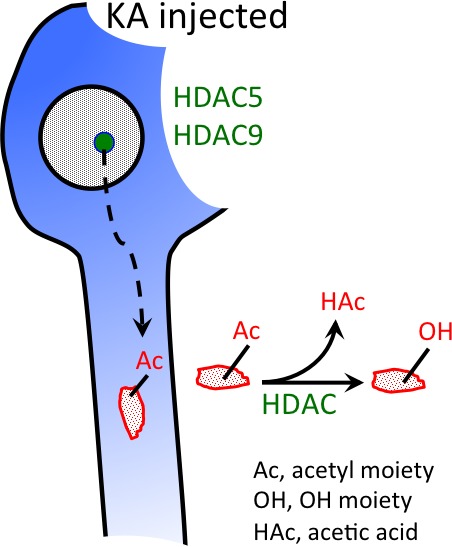

HDAC 5 and HDAC 9 expression is highly increased in granule cells of the KA‐injected hippocampus and parallels granule cell dispersion. Both HDACs are thought to be targeted to the cytoplasm and to act there by deacetylating cytoplasmatic (e.g. cytosceleton‐related) proteins.

**Abbreviations used**
AMPA: alpha‐amino‐3‐hydroxy‐5‐methyl‐isoxazole‐propionic acid
GluA2: AMPA receptor subunit 2
HDAC: histone deacetylase
HPD: hippocampal paroxysmal discharge
KA: kainic acid
NSE: neuron specific enolase
TLE: temporal lobe epilepsy

Temporal lobe epilepsy (TLE) comprises about 30% of all epilepsies and about half of the TLE patients are resistant to antiepileptic drug treatment (Fisher *et al*. 1998). The most common pathology underlying TLE is Ammon's horn sclerosis characterized by cell losses in the hilus of the dentate gyrus and in hippocampal sectors CA1 and CA3, while other brain areas are less affected (Babb *et al*. 1984). In about half of the patients with hippocampal sclerosis, a broadening of the granule cell layer or even a double granule cell layer is observed, termed granule cell dispersion (Houser 1990; Haas *et al*. 2002). In general, TLE develops after an initial insult, which can be early febrile seizures, tumors, or traumatic brain injury. It may take, however, years from the initial insult until the first clinical symptoms of TLE develop. This time window is termed ‘silent phase’. Changes in the molecular biology or morphology of some brain circuitries may develop during this period, ultimately leading to the manifestation of epilepsy. It is associated with pronounced changes in the expression of many neuropeptides and proteins like cyclooxygenase, glutamate decarboxylases, the T‐type calcium channel Cav3.2, neuropeptide Y, dynorphin, and many others (Forstermann *et al*. 1982; Houser *et al*. 1990; Furtinger *et al*. 2001; Becker *et al*. 2008; Sperk *et al*. 2012). The underlying mechanisms leading to changes in the expression of these proteins and their function for the development of epilepsy are still poorly understood.

Animal models investigating the process of epileptogenesis are mimicking the initial epileptogenic insult. This may include induction of an initial status epilepticus, e.g. by injection of convulsive toxins like kainic acid (KA) or pilocarpine (Turski *et al*. 1983; Sperk 1994), setting an injury to the cerebral cortex (Pitkanen and McIntosh 2006), or triggering febrile seizures in infant rodents (Toth *et al*. 1998). Recently, a mouse model has been developed based on the unilateral intrahippocampal injection of KA triggering an initial status epilepticus with subsequent spontaneous seizures (Bouilleret *et al*. 1999; Riban *et al*. 2002). Most interestingly, these mice develop also granule cell dispersion in the KA‐injected hippocampus and the model has been used to study this phenomenon. It appears that the extracellular matrix molecule reelin also guides the correct lamination of the adult granule cell layer in the dentate gyrus and that a loss of reelin may be causative for granule cell dispersion in this animal model and in human TLE (Suzuki *et al*. 2000; Haas *et al*. 2002; Heinrich *et al*. 2006; Haas and Frotscher 2010). On the other hand, systemic injection of pilocarpine results in development of spontaneous seizures without granule cell dispersion.

Possible molecular mechanisms contributing to epileptogenesis include inflammatory and neurodegenerative processes, plastic changes, or altered signaling, e.g. by down‐regulation of alpha‐amino‐3‐hydroxy‐5‐methyl‐isoxazole‐propionic acid (AMPA) receptor subunit GluA2 (Grooms *et al*. 2000; Gorter *et al*. 2006) or up‐regulation of the T‐type calcium channel α1‐subunit Ca*v*3.2 (Becker *et al*. 2008). In addition, a variety of potentially anticonvulsive mechanisms are activated to presumably counteract epileptogenesis. These include increased expression of the GABA‐synthesizing enzyme glutamate decarboxylase or of anticonvulsive neuropeptides such as neuropeptide Y or dynorphin causing augmented inhibition (Houser *et al*. 1990; Furtinger *et al*. 2001; Pirker *et al*. 2009; Sperk *et al*. 2012).

The prominent epilepsy‐triggered changes may be guided by epigenetic mechanisms (Hwang *et al*. 2013; Kobow and Blumcke 2014). These comprise modifications of DNA and histones. Histone modifications include acetylation, methylation, phosphorylation, and other modifications of specific amino acids in the N‐terminal tail of histones (Khorasanizadeh and Ostankovitch 2014). Some modifications may activate gene expression, while others inhibit. Among these modifications, histone methylation and acetylation have been extensively studied in recent years. Histone acetylation is catalyzed by histone acetyltransferases and reversed by histone deacetylases (HDACs). Acetylation of histones generally induces a more permissive (open) state of chromatin and increased gene expression while deacetylation does the reverse (Kimura *et al*. 2005). In humans and rodents, four major classes of HDACs have been characterized (Chuang *et al*. 2009). Class I HDACs include HDAC1, 2, 3, and 8. Class II HDACs are divided into class IIa HDACs including HDAC4, 5, 7, and 9, and class IIb enzymes including HDAC6 and 10. Class III HDACs are referred to as sirtuins based on their homology to the yeast HDAC Sir2. Because of its distinct structure, HDAC11 has been attributed to a new class, class IV (Voelter‐Mahlknecht *et al*. 2005). Class I, II, and IV are zinc dependent; sirtuins are regulated by NAD^+^. Class I HDACs are generally restricted to the nucleus where they impose transcriptional control, whereas class IIa HDACs transit the nuclear membranes and enter the cytoplasm in a process mediated by phosphorylation (Yang and Grégoire 2005) and HDAC6 is primarily cytoplasmic (Kawaguchi *et al*., 2003). Although Class I HDACs may be primarily (although not exclusively) involved in transcriptional regulation, class II HDACs may also target cytoplasmatic targets. Thus, upon peripheral nerve injury, HDAC5 has been shown to deacetylate tubulin and by this to guide axonal regrowth (Cho and Cavalli 2012; Cho *et al*. 2013).

In a recent study, we demonstrated that rapid decreases in the expression of class I HDACs occur after acute KA‐ and pilocarpine‐induced status epilepticus and may contribute to the development of epilepsy (Jagirdar *et al*. 2015). Here, we report changes in the expression of class II HDACs in the same mouse models of epilepsy.

## Methods

### Animals

All animal experiments were conducted according to national guidelines and European Community laws and were approved by the *Committee for Animal Protection* of the Austrian Ministry of Science. In total, we used 91 adult, 12–16 weeks old male C57Bl6/N mice (25–30 g; Charles River Laboratories, Sulzberg, Germany) for local injection of KA, 24 adult male mice for injection of pilocarpine, and 33 saline injected mice. The animals were housed in groups of 3–5 in Sealsafe^™^ IVC cages (Techniplast GmbH, Hohenpeissenberg, Germany) under standard laboratory conditions (12/12 h light/dark cycle). They had access to food and water *ad libitum*.

### Kainic acid model of TLE

#### Intrahippocampal injection of KA and placement of electrodes

Surgical procedures have been described in detail previously (Weiergräber *et al*. 2005; Drexel *et al*. 2012b). KA (350 pmol in 70 nL in a solution of 5 mM in 0.9% NaCl, pH 7.0; Ascent Scientific Ltd., North Somerset, UK) was injected unilaterally into the stratum lacunosum/radiatum CA1 of the dorsal hippocampus at the following stereotactic coordinates from bregma): posterior, −1.9 mm; lateral, ± 1.6 mm; ventral, −1.9 mm. Immediately after KA injection, biopotential transmitters (TA10EA‐F20; Data Sciences International, Arden Hills, MN, USA) were implanted in a subcutaneous cavity at the abdomen and the recording electrode was placed epidurally through the same hole as for infusion of KA. The reference electrode was placed on the contralateral side. They were fixed to the scull by embedding them together with two stainless steel screws placed in an epidural position in dental cement. The scalp skin was closed and the mice were subjected to post‐surgical care as described in detail previously (Drexel *et al*. 2012b).

### EEG‐ telemetry and video monitoring

Mice were single housed in single ventilated cages. EEGs were recorded using an EEG‐telemetry system (Dataquest A.R.T., Data Acquisition 4.0 for telemetry systems; Data Sciences International) and behavioral responses were video monitored using infrared sensitive Axis 221 network cameras (Axis Communications AB, Lund, Sweden) as described in detail previously (Drexel *et al*. 2012b).

EEGs were recorded at a sampling rate of 1000 Hz with no *a priori* filter cutoff. They were started 30 min after surgery and inspected visually. Seizures defined by EEG were investigated for a behavioral correlate by inspecting the synchronized video recordings. Seizure ratings were defined in the following way: stage 1, staring, arrest, chewing; stage 2, unilateral or bilateral tonic movements/seizure; stage 3, rearing without falling; stage 4, rearing with falling, limbic seizures; stage 5, death. Spectrograms of the EEG traces (fast Fourier transformation, FFT segment length of 4 s) were generated using the SIGVIEW software package (v. 2.6; SignalLab, Pforzheim, Germany). EEG seizures highly correlated with generalized behavioral seizures. We therefore assume that seizures were spreading from the injected hippocampus to the contralateral side and affected the overlaying cortical areas. This is supported by the pronounced changes in expression of class I and IV HDAC mRNAs in both hippocampi. Furthermore, although using an epidural recording electrode, we well detected the frequent hippocampal paroxysmal discharges (HPD) originating from the injected hippocampus. These have been shown to spread to the contralateral side and are even reduced after dentate gyrus transections (Pallud *et al*. 2011).

### Pilocarpine model of TLE

For identifying changes in HDAC expression specifically related to seizure induction and epileptogenesis and not to particular effects of the KA model (in particular granule cell dispersion), we investigated changes in HDAC expression also in the pilocarpine model of epileptogenesis (Cavalheiro *et al*. 1996). These mice, similar to the KA‐injected mice, show a status epilepticus and epileptogenesis, but no granule cell dispersion (Cavalheiro *et al*. 1996). Male mice (20–25 g) were injected with *N*‐methyl scopolamine (170 mg/kg in saline; s.c.) and after 20 min with pilocarpine (Pitsch *et al*. 2007). All mice rapidly developed seizures leading to a status epilepticus and were treated with 4 mg/kg i.p. diazepam (Gewacalm; Nycomed, Linz, Austria) 40 min after the first stage 3 seizure to suppress the status epilepticus. Control mice were injected with *N*‐methyl scopolamine followed by saline injections. Seizures were rated according to the same rating scale given above.

### Tissue preparation

KA‐injected mice were killed in a CO_2_ chamber at different time intervals including short‐time intervals, 2, 4, 6, 12, 24, and 48 h and long‐time intervals 7, 14, and 28 days. In the pilocarpine experiment, mice were killed 4 h, 24 h, and 28 days after pilocarpine injection. Mice were then immediately decapitated, the brains were removed and snap frozen in isopentane (−70°C for 3 min). Brains were then kept at −70°C in open tubes for 24 h to allow the isopentane to evaporate. They were then stored in sealed vials. Coronal 20 μm sections were cut with a cryotome (HM 560 M; Microm GmbH, Walldorf, Germany) and thaw‐mounted onto gelatin‐coated slides. The sections were stored at −20°C until further use.

### *In situ* hybridization

Oligonucleotides complementary to the mRNA sequences of the individual HDACs were designed and obtained HPLC purified from Microsynth (Balgach, Switzerland). They are listed in Table S1.

*In situ* hybridization was performed as described previously in detail (Furtinger *et al*. 2001). Series of sections containing two anatomically matched sections of each animal of the respective experiment (from controls, and KA and pilocarpine treated mice, respectively) were processed concomitantly. Oligonucleotides (2.5 pmol, Table S1) were 3′ end labeled with [^33^P]α‐dATP (75 μCi; 3000 Ci/mmol, SCF‐203; Hartmann Analytic, Braunschweig, Germany) and terminal transferase (Roche Diagnostics, Basel, Switzerland), as described previously in detail (Drexel *et al*. 2012a). Hybridization was performed in 50% formamide, 4× saline sodium citrate buffer (SSC) (1× SSC is 150 mM NaCl, 15 mM sodium citrate, pH 7.2), 500 μg/mL salmon sperm DNA, 250 μg/mL yeast tRNA, 1× Denhardt's solution (0.02% Ficoll, 0.02% polyvinylpyrrolidone, and 0.02% bovine serum albumin), 10% dextran sulfate, and 20 mM dithiothreitol (all from Sigma, St Louis, MO, USA) at 42°C for 18 h. The slides were washed stringently (50% formamide in 1× SSC, 42°C), and then rinsed in water briefly, followed by 70% ethanol, and dried. They were then exposed to BioMax MR films (Amersham Pharmacia Biotech, Buckinghamshire, UK) together with [^14^C]‐microscales for 14–28 days. The films were developed with Kodak D19 developer. Sections were counter‐stained with cresyl violet, dehydrated, cleared in butyl acetate, and covered using Eukitt (Merck, Darmstadt, Germany). To test the specificity of the oligonucleotide probes, two different probes were used for HDACs 4, 6, and 9 separately and in combination (see Table S1) at different stringencies of the SSC washing buffer (0.5, 1, 2× SSC). The optimal condition was 1× SSC yielding the same distribution for each HDAC species. The label was displaced by excess (50 pmol) of unlabeled probe.

### Quantitative evaluation

Quantitative evaluation was done on digitized images of the autoradiograms (eight bit digitized picture, 256 gray values) as described previously (Tsunashima *et al*. 1997). Gray values were measured by the public domain program ImageJ 1.38x (NIH, Bethesda, MD, USA; 255 = white; 0 = black) and converted to relative optical density (ROD). [^14^C]‐micro‐scales (Amersham Pharmacia Biotech, Amersham, UK) were exposed together with the slides to film. They were used to draw a standard curve and to verify that ROD values were within the linear range of this standard curve.

### Nissl stain

For adjusting the levels of brain sections obtained from different mice, every tenth section collected was Nissl stained (Franklin and Paxinos 2008). Briefly, sections were mounted on slides, dried, dehydrated in graded ethanol, and transferred to butyl acetate. After rehydration in graded ethanol/H_2_O, sections were incubated in 0.5% cresyl violet for 5 min and again dehydrated in graded ethanol, transferred to butyl acetate, and coverslipped with Eukitt (O. Kindler GmbH, Freiburg, Germany). For evaluating neurodegeneration, densities of hippocampal cell layers were determined at different intervals after intrahippocampal KA injection and after pilocarpine‐induced status epilepticus. Nissl‐stained sections were scanned and gray values of individual hippocampal cell layers were determined using the ImageJ program and converted to ROD. The extent of granule cell dispersion was estimated similarly determining the area of granule cells using ImageJ. The data are expressed as % of controls ± SEM. Statistics: anova with Dunnett's *post hoc* test. *p < 0.05, **p < 0.01;***p < 0.001.

### Statistical analyses

We performed three independent experiments. They were first evaluated separately. Since no significant differences were observed between individual experiments we pooled the %‐values for each interval. The total numbers of mice in the KA experiment were: Controls, 23; 2 h, 12; 4 h, 9; 6 h, 10; 12 h, 4; 24 h, 4; 48 h, 3; 7 days, 6; 14 days, 5; 28 days, 7 mice. In the pilocarpine experiment, the following numbers of mice were used: Controls, 10; 4 h, 9; 24 h, 5; 28 days, 4 mice. ROD values were calculated as % of the mean ROD of controls. Data are shown as mean ± SEM. Statistical analysis was done using the GraphPad Prism program (v 5.0d; GraphPad Software Inc., La Jolla, CA, USA). Relative optic densitometry data were analyzed by one‐way anova with *post hoc* Dunnett test. FFT‐derived results were analyzed by two‐way anova and Bonferroni *post hoc* test.

## Results

### Kainic acid model

#### EEG and motor seizures

**Figure 1:**
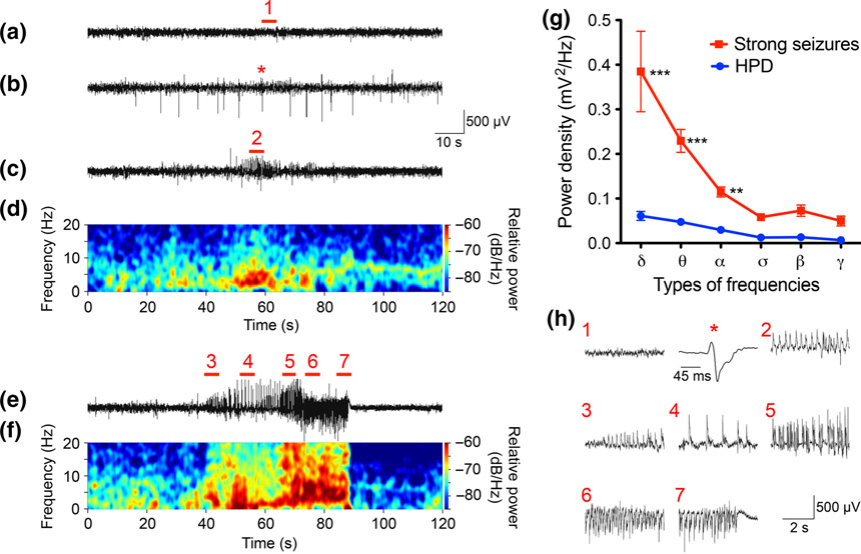
EEG activity induced by intrahippocampal injection of kainic acid (KA). Baseline EEG‐recording in a vehicle injected mouse (a); spike series (b), a hippocampal paroxysmal discharge (c) and a strong spontaneous seizure (e) in KA‐injected mice. In (d) and (f) power spectra from traces in (c) and (e) are shown. (g, h) depicts magnified details from (a, b, c, e) as indicated there with red numbers 1–7 and an asterisk (note the different time scale in h) for a spike‐wave discharge in (b). In (g) power spectrum analysis of strong seizures and hippocampal paroxysmal discharges (HPD) is shown. Frequency distribution analysis revealed significantly elevated power density of δ (delta, 0.5–4 Hz), θ (theta, 4–8 Hz) and α (alpha, 8–12 Hz) frequencies but not for σ (sigma, 12–16 Hz), β (beta, 16–24 Hz) and γ (gamma, 41–99 Hz) frequencies in strong seizures compared to HPD. The data are expressed as mean ± SEM value for 30 strong seizures and 50 HPDs from four animals. **p < 0.01, ***p < 0.001, two‐way anova followed by Bonferroni *post hoc* test.

Telemetrically recorded EEGs and behavior of the KA injected mice were in accordance with previously published data (Bouilleret *et al*. 1999; Riban *et al*. 2002; Pallud *et al*. 2011) and have been described for our mouse cohorts in detail elsewhere (Jagirdar *et al*. 2015). Briefly, as summarized in Fig. 1, EEG changes were characterized by three types of seizure‐like events, (i) the acute status epilepticus, (ii) frequent HPD, and (iii) rare spontaneous strong seizures. The EEG of KA‐injected mice showed also frequently occurring spikes and spike trains (Fig. 1b and h).

### Status epilepticus

The status epilepticus was characterized by frequent episodes of seizures (about 10/h), lasting for about 2 min each. EEG discharges associated with status epilepticus lasted 6–12 h and became significantly reduced in amplitude and frequency thereafter. Motor seizures were suppressed by the initial anesthesia and were observed to be rare and mild in the video recordings.

### Hippocampal paroxysmal discharges

Spike/wave discharges during status epilepticus turned into short HPD after about 48 h (Fig. 1c, d and h). These were characterized by short periods of increased EEG activity, but without obvious behavioral changes and were not followed by a postictal depression of the EEG amplitude (Pallud *et al*. 2011). As determined in this animal model previously (Pallud *et al*. 2011), the mean rate of HPD was around 50 per 24 h; the mean amplitude 0.77 ± 0.02 mV and their mean duration was around 18 s, and they were observed at the same rate during the entire recording period of 28 days (Jagirdar *et al*. 2015).

### Spontaneous strong EEG seizures

Spontaneous strong EEG seizures developed 36 h after KA injection (Fig. 1e, f and h). As shown previously (Riban *et al*. 2002; Pallud *et al*. 2011) they were characterized by high‐amplitude oscillations (1.44 ± 0.058 mV) with a mean duration of about 45 s. Spontaneous strong seizures progressed gradually, lasting for more than 30 s, and commonly returned to baseline directly followed by postictal flattening of the EEG signal below the baseline (postictal depression, Fig. 1e, f and h). They occurred at a rate of about 1.5 per 24 h. Power analysis revealed a considerably higher power density of strong seizures in the low frequency bands (α, θ, δ) than HPDs (Fig. 1g).

### Histopathology of kainic acid model

**Figure 2:**
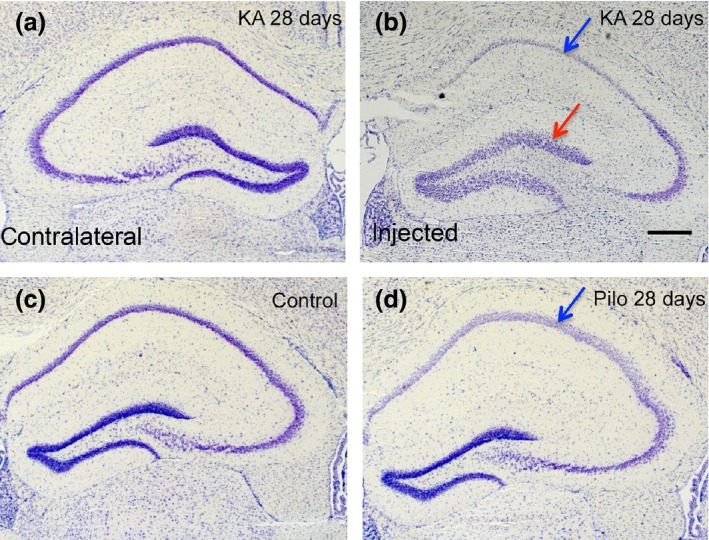
Nissl staining after status epilepticus induced by unilateral intrahippocampal injection of kainic acid (KA) and by pilocarpine. (c) control hippocampus; (a) and (b) show the contralateral and ipsilateral side 14 days after local KA injection. Note the pronounced granule cell dispersion in the injected hippocampus (red arrow in b) with neurodegeneration in sectors CA1 (blue arrow in b) and CA3. (d) 28 days after i.p. pilocarpine; note the neurodegeneration in sectors CA1 (blue arrow in d) and CA3 of the hippocampus. Scale bar in (b): 400 μm.

In our study, we used lower doses of KA (350 pmol compared to 1 nmol) than in previous studies (Bouilleret *et al*. 1999; Riban *et al*. 2002; Heinrich *et al*. 2006; Pallud *et al*. 2011). This resulted in less neurodegenerative changes although similar behavioral responses and similar granule cell dispersion were observed (Fig. 2b). The injection was placed unilaterally into the area of the stratum radiatum/stratum lacunosum‐moleculare of sector CA1. At the injection site, losses in CA1 pyramidal neurons and of interneurons were observed at all intervals investigated. These were not seen in the contralateral hippocampus (Table S2). Thus, evaluations of HDAC mRNAs were done there. At the early intervals (4 and 12 h after KA injection), the granule cell layer was not affected (not shown). At later intervals (24 h), we observed modest and thereafter (14 and 28 days after KA injection) pronounced granule cell dispersion in the injected hippocampus (Fig. 2d). The contralateral hippocampus showed no significant signs of cell loss. Also, no neurodegenerative changes were seen in other brain areas such as the amygdala or entorhinal cortex.

### Distribution of HDAC mRNAs in the control hippocampus

**Figure 3:**
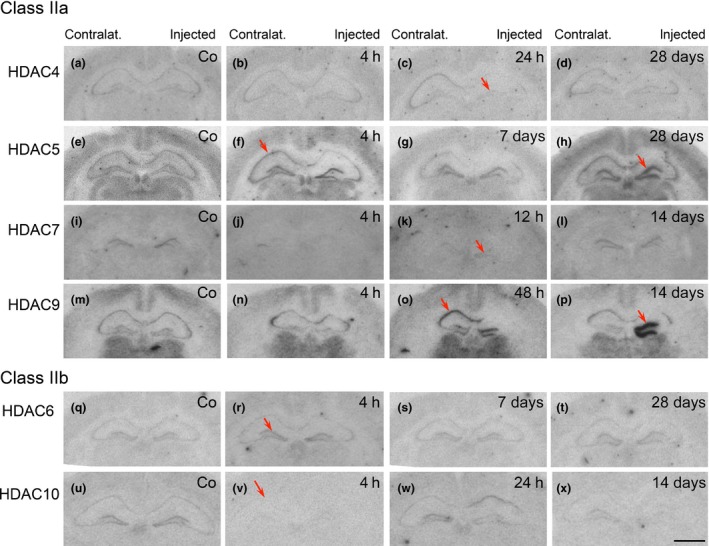
Expression of class IIa histone deacetylase (HDAC)4, 5, 7, and 9 and class II b HDAC6 and 10 mRNAs at various time points after unilateral injection of kainic acid (KA) into the hippocampus. The figure shows autoradiographs after *in situ* hybridization with radiolabeled antisense probes for the individual HDACs, from controls (Co) and at selected time points after injection of KA into the right hippocampus. Note signs of neurodegeneration in the right, injected hippocampus. Arrows indicate: (f) and (h) increased expression of HDAC5 in the pyramidal cell layer 4 h after KA and in the granule cell layer of the injected hippocampus after 28 days, respectively; (j, k) decreased expression of HDAC7 in the granule cell layer 4 and 12 h after KA, respectively; (c) decreased expression of HDAC4 in granule cells 6 h after KA; (o, p) markedly increased expression of HDAC9 in the pyramidal cell layer after 48 h and in the granule cell layer of the KA injected hippocampus after 14 days. Increased and decreased expression of HDAC6 and HDAC10, respectively in the granule cell layer 4 h after KA injection (r, v). Compare quantitative evaluation shown in Fig. 4 for class IIa HDAC mRNAs. Scale bar in (x) (for a–x): 1 mm.

All HDAC mRNAs, except those for HDAC7 were well detected throughout the hippocampal formation (Figure S1, Figs 3 and 5). The relative intensities of expression as well as the distribution of individual HDAC mRNAs compared well to those reported for the rat hippocampus recently (Broide *et al*. 2007). Expression of HDAC5 appeared to be considerably stronger than that of class IIa HDACs 4, 7, and 9 and class IIb HDAC mRNAs 6 and 10. The patterns of distribution between individual HDAC species were different. Thus, HDAC 4, 5, and 6 mRNAs seemed to be equally distributed in all principal cell layers of the hippocampus. HDAC9 mRNA was more concentrated in hippocampal sectors CA1 and CA3 than in dentate granule cells. HDACs 4, 5, 6, and 9 were also expressed in other brain areas of the same section notably in the cerebral cortex. Class IIa HDAC 5 and 9 mRNAs were especially prominent in thalamic nuclei. The distribution of class I and IV HDACs in control mice has been reported in our accompanying paper (Jagirdar *et al*. 2015).

### Changes in expression of HDAC mRNAs expression during epileptogenesis in the kainic acid model

To differentiate between direct effects of KA in the injected hippocampus and epilepsy‐related changes, we investigated the expression of HDAC mRNAs in the granule cell layers of the KA‐injected and the contralateral dentate gyrus. Changes in HDAC expression in the pyramidal cell layers were investigated in sectors CA1 and CA3 in the contralateral hippocampus, which was not affected by neurodegeneration induced by the local KA injection.

### Class IIa: HDAC 5 and 9 mRNAs

#### Granule cell layers

**Figure 4:**
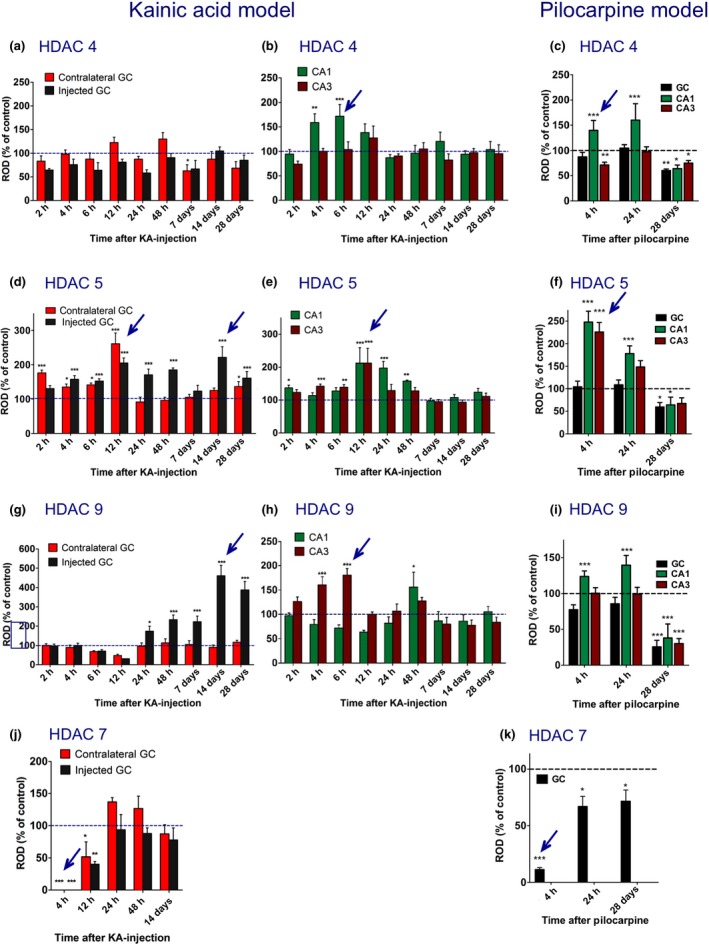
Time course of changes in expression of class IIa histone deacetylase (HDAC)4, 5, 7, and 9 mRNAs in the intrahippocampal kainic acid (KA) and in the pilocarpine model. The figures show changes in the mRNA expression as relative optic densities expressed as % of controls ± SEM. The left column (a, d, g, j) depicts changes in mRNA expression in the granule cell layer of the dentate gyrus (GC) in the KA model comparing the injected (black bars) with the contralateral side (red bars). The middle column (b, e, h) shows changes in the pyramidal cell layers (green bars, CA1; brown bars, CA3) contralateral to the injection side, which is not affected by cell loss upon local injection of KA (Table S2). The right column (c, f, i, k) depicts changes in the pilocarpine model (black bars, granule cell layer; green bars and brown bars, CA1and CA3 pyramidal cell layers, respectively). The values for the sector CA1 were corrected for cell losses in this area (Table S3). Note that HDAC5 and 9 mRNA levels were decreased throughout the hippocampus 28 days after pilocarpine‐induced status epilepticus (f, i), whereas they were increased in the granule cell layer of the KA injected hippocampus (d, g), coinciding with massive granule cell dispersion (see Fig. 2). Also, late (28 days) decreases in HDAC4, 5, and 9 in pyramidal cell layers (c, f, i) oppose the lack of changes in the KA model at this interval (b, e, h). For HDAC,7 mRNA reliable data were only obtained for dentate granule cells. They indicate decreased mRNA expression during the early status epilepticus in both models (j, k). Arrows indicate prominent changes in HDAC mRNA expression. Numbers of mice for the KA model were: Controls, 19–22; 2 h, 6; 4 h, 9; 6 h, 10; 12 h, 4; 24 h, 4; 48 h, 3; 7 days, 4–6; 14 days, 4–5; 28 days, 7. For the pilocarpine model: Controls, 10; 4 h, 8; 24 h, 5; 28 days, 4. Statistical analysis was done by one‐way anova with Dunnett's *post hoc* test. *p < 0.05, **p < 0.01, ***p < 0.001 versus controls.

Expression of HDAC 5 and 9 mRNAs was most prominently changed. HDAC5 expression was altered in three phases: During ongoing EEG status epilepticus (2–12 h after KA injection), HDAC5 was rapidly increased by about 30–80% both in the granule cell layer of the injected and of the contralateral hippocampus at the 2 h interval and reached 205 ± 14.56% of controls (*p* < 0.001) at the injection site and 261 ± 31.36% of controls (*p* < 0.001) on the contralateral side 12 h after KA injection (Figs 3f and 4d). During the subsequent phase of recovery (24–48 h after KA injection), HDAC5 mRNA levels remained markedly increased (up to 185.2 ± 5.58% of controls; *p* < 0.001) at the injection site, but returned to control levels on the contralateral side (Fig. 4d). At the 7 days interval, HDAC5 mRNA expression was normalized in both hemispheres (Fig. 3g) and then increased again at the late intervals, especially in the granule cell layer of the injected side (Figs 3h and 4d; 221.4 ± 30.67% of controls, *p* < 0.001 after 14 days) when spontaneous EEG and motor seizures were recorded.

Changes in HDAC9 mRNA expression in granule cells of the dentate gyrus were even more conspicuous than those of HDAC5 mRNA. Compared to the expression of other class II HDACs and to that in the pyramidal cell layer, HDAC9 mRNA levels were low in dentate granule cells of controls, possibly indicating a minor role in the regulation of gene expression under physiological conditions (Fig. 3m). Six to 12 h after induction of the status epilepticus HDAC9 mRNA levels declined even further (Fig. 4g; statistically not significant). Thereafter, we observed (only) in the KA injected granule cell layer marked increases in HDAC9 mRNA expression amounting 460.7 ± 54.33% (*p* < 0.001) of controls at the 14 days interval (Figs 3o,p and 4g).

#### Sectors CA1 and CA3 contralateral to the injection of KA

In the pyramidal layers of both sectors, HDAC5 mRNA levels were markedly increased after 12 h (Fig. 4e; CA1: 212.1 ± 46.87%, *p* < 0.001; CA3: 212.1 ± 44.61%, *p* < 0.001). This increase rapidly faded away in the sector CA3, and somewhat more slowly in sector CA1 where it was still significantly increased after 24 h (Fig. 4e; 197.4 ± 19.75%; *p* < 0.001) and 48 h (Fig. 4e; 157.8 ± 2.82%; *p* < 0.01).

At the 48 h interval, HDAC9 mRNA expression was increased in the pyramidal cell layer (Fig. 4h; CA1, 152.1 ± 18.87%, *p* < 0.05 and CA3, 128.3 ± 5.60% vs. controls).

### Class IIa HDAC 4 and 7 mRNAs

HDAC 4 mRNA expression was faint and difficult to evaluate. It appeared to be modestly (not significantly) decreased in the granule cell layer of the injected hippocampus 2 h to 7 days after KA injection (Fig. 4a). It was, however, significantly increased in sector CA1, contralateral to the injection, after 4–6 h (Fig. 4b; 172 ± 23.89%; *p* < 0.001), with no change in sector CA3. HDAC 7 mRNA was only detected in the granule cell layer. It virtually disappeared there on the ipsilateral and contralateral side at 4 h (Figs 3j and 4j). After 12 h, HDAC 7 mRNA levels were still decreased in most mice (Figs 3k, and 4j; mean: 40.4 ± 3.96%, *p* < 0.01, and 51.7 ± 23.03% of control, *p* < 0.05, ipsilateral and contralateral, respectively), but recovered after 24 h and thereafter (Figs 3l and 4j).

### Class IIb HDACs 6 and 10

HDAC6 mRNA was only weakly expressed in the hippocampus (Fig. 3q). We observed transient increases in the granule cell layer contralateral to the KA injection (Figure S2a; 145.6 ± 16.58% of controls; *p* < 0.01) and in pyramidal cell layers of sectors CA1 and CA3 12 h after KA injection (Figure S2b; CA1, 192.6 ± 27.78%; *p* < 0.001; CA3: 145.1 ± 18.36%, *p* < 0.05). Expression of HDAC10 was also faint (Fig. 3u). It revealed bilateral decreases in the granule and pyramidal cell layers in its levels 4 h after KA injection (Fig. 3v and Figure S2d and e).

### Expression of β‐actin and neuron specific enolase

To investigate on ‘unspecific’ regulation of mRNAs, we also assessed expression of β‐actin and neuron specific enolase (NSE) mRNA in sections of each interval after KA injection. We observed no obvious changes in β‐actin and NSE mRNA expression at any time point (Figure S3).

### Pilocarpine model

#### Behavior and neuropathology

As described by others before (Pitsch *et al*. 2007), pilocarpine induced a status epilepticus characterized by tonic–clonic seizures and limbic convulsions with rearing and falling over in all mice investigated. Behavioral status epilepticus occurred 20–30 min after pilocarpine injection and was interrupted by injection of diazepam 90 min after the first stage 3 seizure. Six of 24 pilocarpine injected mice died. Histopathological changes were characterized by 22–24% losses of CA1 pyramidal neurons seen at the 4 h and 28 day intervals. No losses were observed in sector CA1 and in the granule cell layer (Table S3) and no granule cell dispersion was observed (Fig. 2d).

### Expression of HDAC mRNAs in the pilocarpine model of TLE

Also in the pilocarpine model of TLE, changes in the expression of individual HDACs followed distinctly different patterns, both in the regional expression and in the temporal patterns. Other than after intrahippocampal injection of KA, changes in the expression of individual transcripts were rather similar in the granule cell layer and in pyramidal cell layers at the different time intervals.

### Class IIa HDAC 4, 5, 7, and 9 mRNAs after pilocarpine‐induced seizures

**Figure 5:**
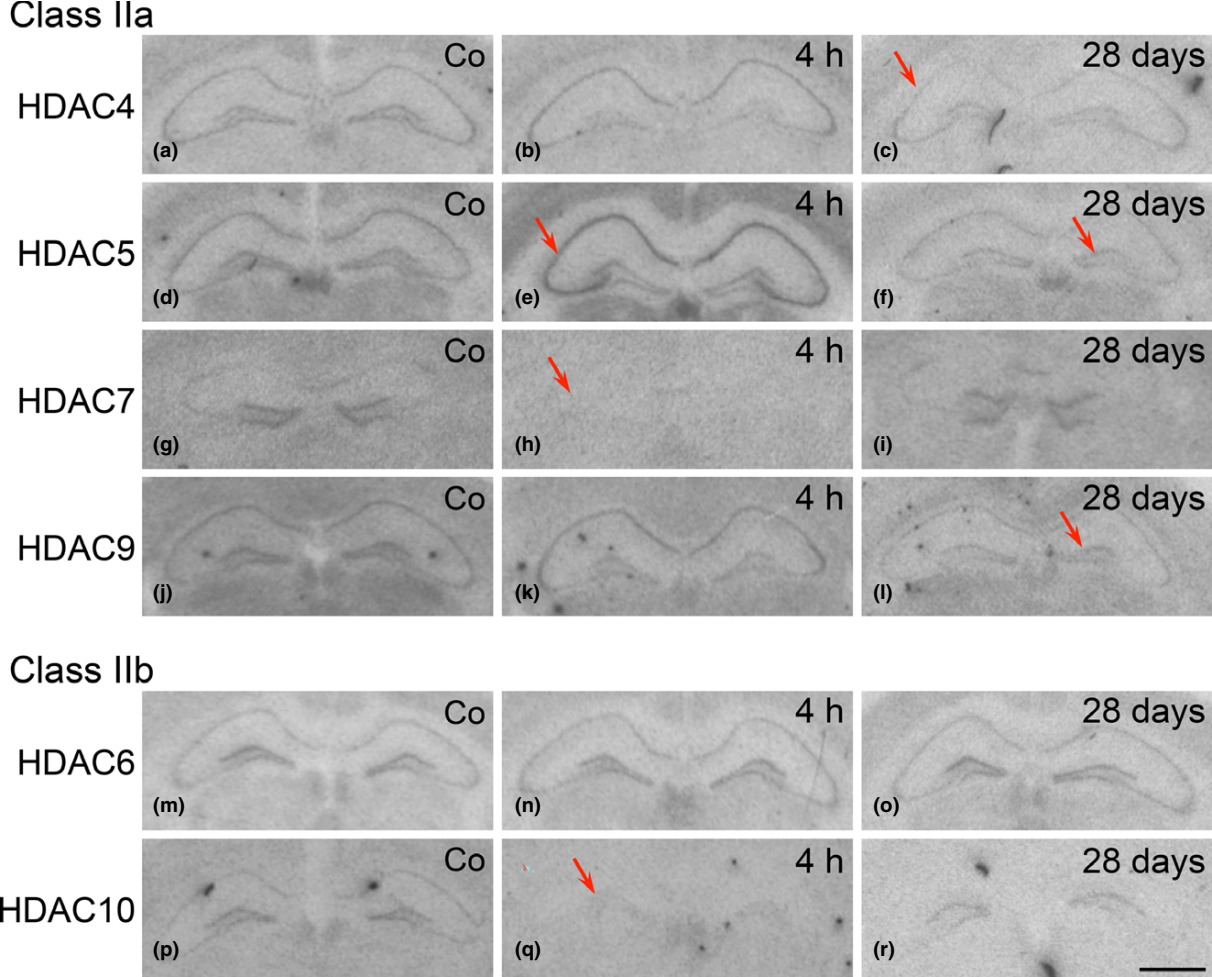
Expression of class IIa and b histone deacetylase (HDAC) mRNAs after pilocarpine‐induced status epilepticus. Shown are photomicrographs from autoradiographs of controls (Co) and selected time points after pilocarpine injection (4 h and 28 days) after *in situ* hybridization with radiolabeled antisense probes for the individual HDACs. Arrows indicate: in (c) decreased expression of HDAC4 in the pyramidal cell layer 28 days after pilocarpine injection; in (e) increased expression of HDAC5 mRNA in the pyramidal cell layer 4 h after pilocarpine; in (f) decreased expression of HDAC5 in the granule cell layer after 28 days (in contrast to the injected hippocampus in the kainic acid model); in (h) decreased expression of HDAC7 throughout the hippocampus after 4 h; in (l) decreased expression of HDAC9 mRNA in the granule cell layer after 28 days; in (q) decreased expression of HDAC10 after 4 h. Scale bar in (r) (for a–r): 1 mm.

HDAC7 mRNA levels decreased 4 h after pilocarpine injection to 11.3 ± 1.69% of controls, *p* < 0.001 (Figs 4k and 5h). At the same time (4 h after pilocarpine), HDAC5 mRNA levels increased in CA1 (248.0 ± 24.21% of control; *p* < 0.001) and CA3 (225.9 ± 21.16% of control; *p* < 0.001) pyramidal cell layers (Figs 4f and 5e). They returned nearly to control levels after 24 h. HDAC 4, 5, and 9 revealed marked decreases in their mRNA levels throughout the granule and pyramidal cell layers at the late interval, 28 days after pilocarpine‐induced status epilepticus (HDAC4, DG: 60.2 ± 2.96% of control, *p* < 0.01; CA3: 74.6 ± 5.11%, *p* < 0.05; CA1: 64.0 ± 7.12%, *p* < 0.05 (Figs 4c and 5c); HDAC5, DG: 59.5 ± 9.85% of control, *p* < 0.05; CA3: 67.3 ± 12.59%, *p* > 0.05; CA1: 64.1 ± 17.34%, *p* < 0.05 (Figs 4f and 5f); HDAC9, DG: 25.8 ± 9.10% of control, *p* < 0.001; CA3: 30.3 ± 7.02%, *p* < 0.001; CA1: 38.0 ± 19.41%, *p* < 0.001 (Figs 4i and 5l).

### Class IIb HDAC 6 and 10 mRNAs after pilocarpine‐induced seizures

HDACs 6 and 10 were only faintly expressed. No significant changes were observed for HDAC6 mRNA expression after pilocarpine‐induced seizures (Figs 5n, o and Figure S2c), whereas HDAC10 mRNA appeared to be down‐regulated in all hippocampal subfields at all intervals (Figs 5q, r and Figure S2f).

### HDAC9 mRNA expression in granule cells in the KA model

#### Correlation of late increases in HDAC9 expression with the extent of granule cell dispersion in the KA model

**Figure 6:**
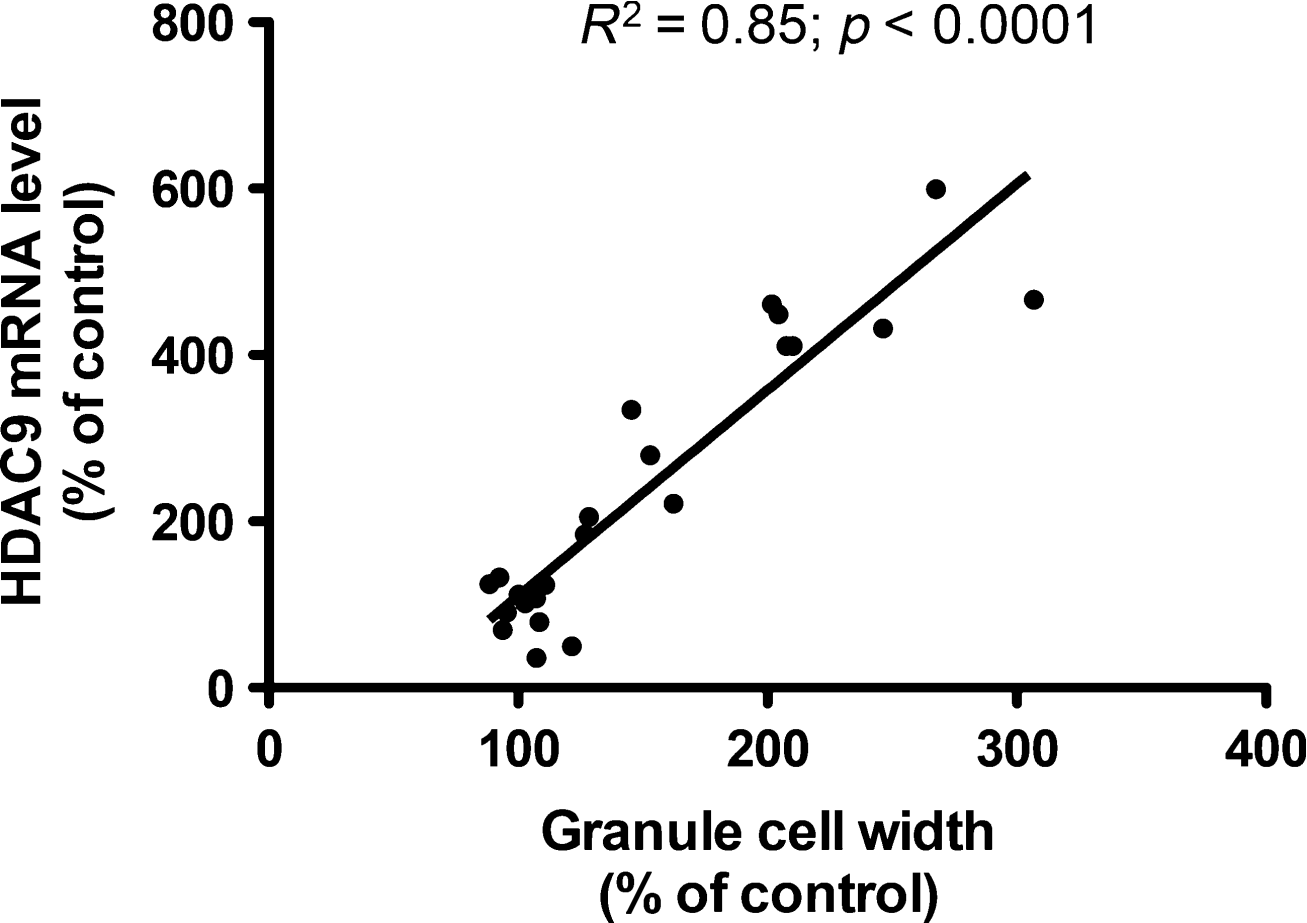
Correlation of histone deacetylase 9 mRNA expression and granule cell dispersion after unilateral hippocampal kainic acid (KA) injection.

A key pathological feature of the animal model based on intrahippocampal injection of KA is the development of prominent granule cell dispersion starting after about 4 days. To investigate whether the increase in HDAC9 mRNA expression may parallel granule cell dispersion, we measured the depth of the granule cell layer and correlated it with the increase in HDAC9 expression. As shown in Fig. 6, we observed a significant correlation (*r* ^2^ = 0.85, *p* < 0.0001) between these two parameters pointing to a possible interdependence of both processes.

### Expression of HDAC9 variants 1 and 2 in dentate granule cells in the KA model

**Figure 7:**
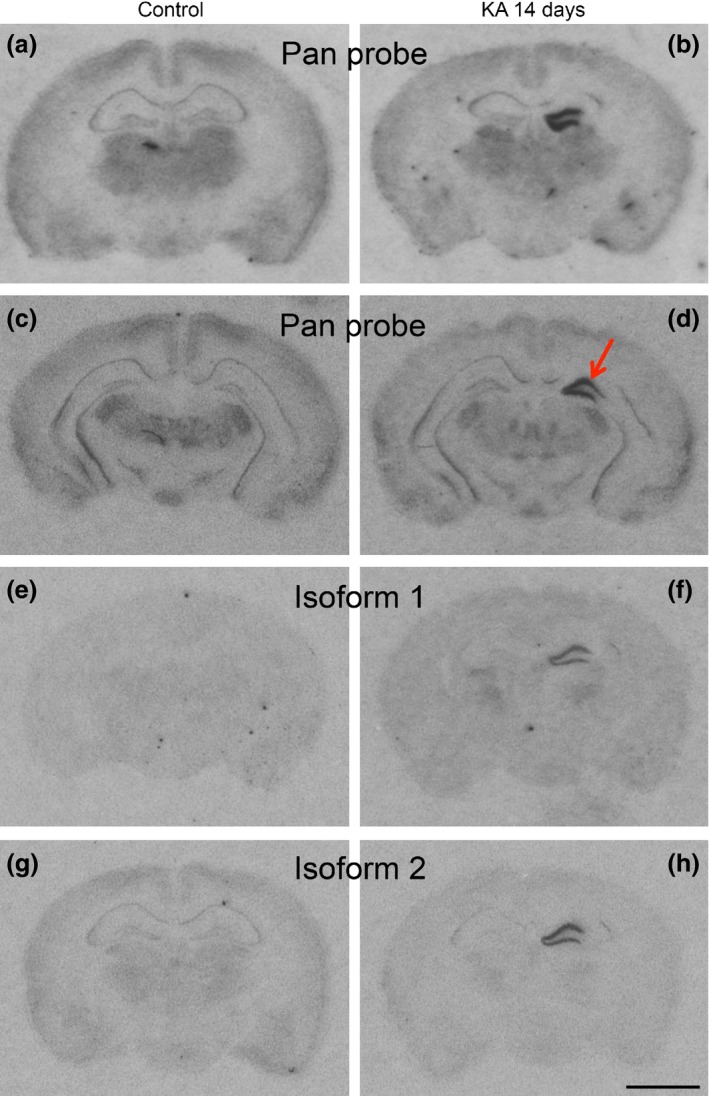
Expression of histone deacetylase (HDAC)9 isoforms at various time points after unilateral hippocampal kainic acid (KA) injection. Photomicrographs of autoradiographs after *in situ* hybridization with a probe for both isoforms (pan probe in a–d), isoform 1 (e, f), and isoform 2 (g, h). Isoform 1 mRNA appeared to be less expressed than isoform 2. Note the strong signals obtained with all three HDAC9 probes in the granule cell layer of the injected but not of the contralateral side. Increased expression of HDAC mRNAs 14 days after KA injection is restricted to the injection site in the dorsal hippocampus and does not extend to the ventral hippocampus (d). Scale bar in (h): 2 mm.

Interestingly, HDAC9 mRNA is expressed in different splice variants (Petrie *et al*. 2003). Our probe equally detected the mRNAs of isoform 1 (1088 amino acids) and isoform 2 (542 amino acids). We therefore designed oligonucleotide probes that specifically recognized only isoform 1 and isoform 2, respectively. As shown in Fig. 7g, the expression pattern of isoform 2 is equal to that obtained with an oligonucleotide probe detecting both isoforms ( Fig. 7a). In contrast, isoform 1 mRNA appeared to be considerably less expressed ( Fig. 7e). However, 14 days after intrahippocampal injection of KA, both mRNA species, the one encoding isoform 1 and that encoding isoform 2, were similarly up‐regulated as the full‐length transcript (Fig. 7b, f and h).

## Discussion

### Seizure behavior of mice in the intrahippocampal KA model

EEG recordings (described for our cohort in Jagirdar *et al*. 2015) were in close agreement with previously published data (Bouilleret *et al*. 1999; Riban *et al*. 2002; Pallud *et al*. 2011). They were characterized by three events, (i) the acute status epilepticus, (ii) frequent HPD, and (iii) rare spontaneous strong seizures. All three events appear to be generated in the hippocampus (Bouilleret *et al*. 1999; Pallud *et al*. 2011) and were well detected by the epidural electrodes placed above the somatosensory cortex. As discussed in more detail elsewhere (Jagirdar *et al*. 2015), the initial KA‐induced status epilepticus was most severe after 4–8 h and lasted up to 36 h. It led over to frequent HPD that have no behavioral equivalent and remained at the same rate during the entire recording period (28 days). The onset of the considerably less frequent spontaneous strong seizures (1–2 per 24 h) is more uniform and faster than in other TLE models (Cavalheiro *et al*. 1996; Pitsch *et al*. 2007; Drexel *et al*. 2012b). Interestingly, full limbic motor seizures (stage 3 and 4) accompanying strong EEG seizures develop with a few days delay. On days 3–5, median seizure rating was 2 and developed to a rating of 4 at days 9–11 (Jagirdar *et al*. 2015). We consider the interval before the onset of the first EEG seizure and the weak motor seizures (days 1–3) as the phase of epileptogenesis. This is also reflected by prominent, but transient changes in expression of class II HDACs at this interval.

Changes in the expression of individual class II HDAC species differed considerably during the acute status epilepticus. Also, the expression patterns of class I and class IV HDAC mRNAs was strikingly different (Jagirdar *et al*. 2015). In contrast to the major class II HDACs (5 and 9), they underwent prominent down‐regulation during the status epilepticus (but similar as class II HDACs 7 and 10). At the phase of epileptogenesis, class I HDACs 1, 2, 3, and 8 were up‐regulated, similar as HDAC5, but not like other class II HDACs. These findings are indicative for specific regulation of each individual HDAC species. The fact that expression of markers presumably not related to epigenetic regulation (β‐actin and NSE) were not significantly changed is supporting this idea.

### Early changes in class II HDAC expression

Although HDAC5 mRNA levels increased throughout the granule and pyramidal cell layers two to 48 h after KA injection, decreased HDAC4 mRNA levels were observed in the injected granule cell layer at the 2 and 24 h intervals. Concomitantly, with the increases in HDAC5 mRNA expression, HDAC4 mRNA levels increased in CA1 and HDAC9 mRNA levels in CA3 pyramidal cells at the 6 and 12 h intervals. Notably, the increased expression of HDAC5 mRNA with a maximum 12 h after KA injection clearly parallels highest seizure activity during the acute status epilepticus, which was maximal after 6–12 h. Since the changes were seen in the contralateral hippocampus, they may be related to the seizure activity and not to neurodegeneration seen only in the KA‐injected hippocampus.

Increased expression of HDAC5 in all principal neurons of the hippocampus (and of HDAC4 in CA1 and of HDAC9 in CA3 pyramidal cells) may result in reduced acetylation of histones H3 and H4 at certain promoters and consequently in down‐regulation of certain genes. In particular, reduced expression of the AMPA receptor subunit GluA2 (but not of GluA1) observed in sectors CA1 and CA3 after KA‐induced status epilepticus may be causative for epileptogenesis (Grooms *et al*. 2000; Gorter *et al*. 2006). GluA2 lacking AMPA receptors mediate an increased Ca^++^ influx upon stimulation and thus increased vulnerability to seizure propagation and excitotoxic brain damage (Grooms *et al*. 2000). And, noteworthy, Huang *et al*. (2002) reported reduced acetylation of the H4 histone at the GluA2 promoter in the hippocampal CA3 sector as early as 3 h after pilocarpine‐induced status epilepticus. Thus, fast (after 2–4 h) up‐regulation of class II HDACs 4, 5, and 9 in pyramidal cells after KA injection lasting up to 12–48 h could contribute to the reduced acetylation of histone H4 at the GluA2 promoter and subsequently reduced GluA2 expression (Huang *et al*. 2002). The HDAC inhibitor trichostatin A prevented and quickly reversed deacetylation of GluR2‐associated histones and blunted seizure‐induced down‐regulation of GluA2 mRNA in CA3, supporting a role of HDACs in the decreased H4 acetylation.

In contrast to HDAC5, expression of class I (HDAC1 and 2) and class IV (HDAC11) HDAC mRNAs is markedly decreased during the initial KA‐induced status epilepticus (Jagirdar *et al*. 2015). These decreases parallel the ones of HDAC7 (class IIb) and may cause a transient increase in the acetylation state of histones and thus can induce augmented expression of certain genes. During the initial status epilepticus, mRNAs encoding *c‐fos, c‐jun*, and brain derived neurotophic factor become strongly up‐regulated (Isackson *et al*. 1991) and seizure‐induced expression of these genes seem to be linked to an enhanced state of acetylation of histone H4 (Sng *et al*. 2006). The HDAC inhibitor trichostatin A augments *c‐fos* and *c‐jun* expression both in control rats and after KA‐induced status epilepticus (Timmusk *et al*. 1993; Sng *et al*. 2005, 2006), which indicates a role of HDACs in the seizure‐induced expression of the immediate early genes.

### Up‐regulation of HDAC5 and HDAC9 in the granule cell layer correlating with granule cell dispersion in the injected hippocampus

One of the most striking observations in our study was the pronounced up‐regulation of the class II HDAC 9 and 5 mRNAs in the granule cell layer of the KA‐injected hippocampus 7–28 days after KA injection. This finding is strikingly contrasted by a marked down‐regulation of both transcripts 28 days after pilocarpine‐induced status epilepticus. These mice do not show granule cell dispersion. We therefore assume that the change in the pilocarpine model may be associated with the chronic epileptic state of the animals. In contrast, over‐expression of HDAC 5 and 9 mRNAs is paralleled by pronounced granule cell dispersion starting to develop around 1 week after KA injection (Bouilleret *et al*. 1999; Suzuki *et al*. 2000). Granule cell dispersion has been first described by Houser *et al*. (1990) in the hippocampus of patients with intractable TLE. It reflects a defect of granule cell lamination in which presumably adult granule cells migrate into the molecular layer of the dentate gyrus (Heinrich *et al*. 2006). Both, in human TLE and after intrahippocampal injection of KA, granule cell dispersion may be caused by a loss of the extracellular matrix molecule reelin and, in the animal model, can be prevented by infusion of recombinant reelin (Muller *et al*. 2009). Interestingly, it has been shown that inhibition of HDACs by trichostatin A results in increased expression of the reelin promoter (Chen *et al*. 2002). Thus, increased expression of HDACs 9 and 5 in granule cells could be related to a decrease in reelin expression in Cajal–Retzius cells (Duveau *et al*. 2011). On the other hand, increased methylation of the reelin promoter correlating with granule cell dispersion has been found in hippocampal specimens from TLE patients (Kobow *et al*. 2009).

It is well known that class IIa HDACs can be exported from the nucleus to the cytoplasm and can shuttle back, a process which is triggered by Ca^++^ and neuronal stimulation (Chawla *et al*. 2003; Schlumm *et al*. 2013). Sugo *et al*. (2010) reported that in cell cultures of mouse cortical neurons, HDAC9 is stimulus dependent exported from the nucleus to the cytoplasm. Transfecting the cells with a plasmid expressing a mutant HDAC9 that cannot be exported from the nucleus to the cytoplasm suppresses dendritic growth of the cells, whereas knock down of HDAC9 with a specific shRNA vector for HDAC9 increased dendritic growth. These experiments suggest that HDAC9 is activity‐dependent exported from the nucleus to the cytoplasm and that it there regulates (suppresses) dendritic arborization. In human TLE, altered dendritic orientation and distribution of dendritic spines in dispersed granule cells has been observed (Freiman *et al*. 2011).

Furthermore, injury of the sciatic nerve can induce export of HDAC5 into the cytoplasm (Cho *et al*. 2013). HDAC5 there deacetylates tubulin and establishes a gradient of deacetylated tubulin guiding the growth cone of the regenerating axon (Cho *et al*. 2013; Cho and Cavalli 2014). A similar mechanism may be possible also for the dispersion of granule cells and their dendrites. The action of HDAC5, however, may be dual. HDAC5 also decreases H3 histone acetylation after injury perhaps also inducing a protective program on the transcriptional level (Cho and Cavalli 2012). However, after axotomy of the optic nerve, a corresponding model of central nerve injury, this mechanism was not observed. An axon growth promoting action has also been observed through activation of HDAC6 in hippocampal neurons presumably also mediated by a prominent tubulin deacetylating activity in the cytoplasm (Tapia *et al*. 2010; Cho and Cavalli 2014). Taken together, there is growing evidence that class II HDACs not only act as HDACs, but are also exported into the cytoplasm where they act as deacetylases on cytoplasmatic proteins. Both mechanisms may be crucial for plastic changes upon injury or nerve stimulation, possibly also in epilepsy‐induced granule cell dispersion. It is worth mentioning that also the sirtuin SIRT1, a protein deacetylase with nuclear and cytoplasmatic functions, is lastingly over‐expressed in the kindling model of epilepsy and in human temporal lobe epilepsy (Chen *et al*. 2013). It is also interesting that concomitantly with granule cell dispersion GluA2 mRNA expression is up‐regulated in granule cells of the KA‐injected hippocampus, although GluA2 is generally down‐regulated in epilepsy (Suzuki *et al*. 2000). Rats do not develop granule cell dispersion upon intrahippocampal KA injection and do not show increased GluA2 expression in granule cells (Suzuki *et al*. 2000). This indicates a close correlation of granule cell dispersion with GluA2 expression.

### Changes in the i.p. pilocarpine model versus the intrahippocampal KA model

Our main purpose in investigating the pilocarpine model concomitantly with the intrahippocampal KA model was to clarify whether the late (14–28 days) increases in HDAC 9 and 5 mRNAs in the granule cell layer of the KA‐injected hippocampus may be initiated by recurrent spontaneous seizures. Since the increases in HDAC 5 and 9 mRNAs are unilateral, this appeared to be unlikely. Furthermore, HDAC 5 and 9 mRNAs (similarly as those for HDACs 4 and 10) became even down‐regulated 28 days after pilocarpine treatment supporting a causative relation between HDAC expression and granule cell dispersion and not with spontaneous seizure activity. There were, however, also striking similarities between the two models, such as the rapid and pronounced down‐regulation of HDAC7 and 10 mRNAs during the status epilepticus and the transient up‐regulation of HDAC5 mRNA 4 and 12 h after the initial status epilepticus, respectively, presumably leading to respective changes in histone acetylation and protein expression. Also, class I HDACs showed marked similarities in their expression patterns during the status epilepticus (after 4 h) and/or at the interval post‐status (after 24–48 h) in both models of status epilepticus‐induced epilepsy (Jagirdar *et al*. 2015).

## Conclusion

In conclusion, expression of different class II HDACs shows highly specific time‐dependent changes in two animal models of TLE. The most striking observation was a markedly increased expression of HDACs 9 and 5 in the granule cell layer 14 days after local injection of KA. Increased expression of HDACs strikingly coincides with prominent granule cell dispersion at this time interval. It is likely that the HDACs are exported from the nucleus into the cytoplasm and exert their deacetylating activity upon cytoplasmatic proteins related to granule cell dispersion. On the other hand, increases in HDAC5 expression and decreases in HDAC7 expression during the acute status epilepticus may have a role in regulating expression of seizure‐related genes by specifically acetylating histones. Our data indicate a potential role of selective HDAC inhibitors for interfering with epileptogenesis.

## Supporting information


**Figure S1.** Expression patterns of mRNAs encoding class II HDACs in control brains.
**Figure S2.** Time course of changes in expression of class IIb HDAC (HDAC 6 and 10) mRNAs in the kainate and pilocarpine model.
**Figure S3.** *In situ* hybridization for β‐actin (A) and neuron specific enolase (NSE, B) mRNAs in a controls (Cont) and 4 h, 6 h, 12 h, 24 h, and 14 days after intrahippocampal KA injection (right hemisphere). Scale bar in B = 2 mm.
**Table S1.** Sequences of oligonucleotide probes.
**Table S2.** Densities of hippocampal cell layers after local intrahippocampal injection of KA.
**Table S3.** Density of hippocampal cell layers after pilocarpine‐induced status epilepticus.
