# Supplementary material for: Expression of class II histone deacetylases in two mouse models of temporal lobe epilepsy
Source: J Neurochem. 2015 Dec 28;136(4):717–30. doi: 10.1111/jnc.13440 (PMC4738395; doi:10.1111/jnc.13440)
Supplement: Supplementary file 1 — Figure S1. Expression patterns of mRNAs encoding class II HDACs in control brains. Figure S2. Time course of changes in expression of class IIb HDAC (HDAC 6 and 10) mRNAs in the kainate and pilocarpine model. Figure S3. In situ hybridization for β‐actin (A) and neuron specific enolase (NSE, B) mRNAs in a controls (Cont) and 4 h, 6 h, 12 h, 24 h, and 14 days after intrahippocampal KA injection (right hemisphere). Scale bar in B = 2 mm. Table S1. Sequences of oligonucleotide probes. Table S2. Densities of hippocampal cell layers after local intrahippocampal injection of KA. Table S3. Density of hippocampal cell layers after pilocarpine‐induced status epilepticus. [file JNC-136-717-s001.docx]

Supporting information for:

**Expression of class II HDACs in two mouse models of temporal lobe epilepsy**

Rohan Jagirdar^a^, Meinrad Drexel^a^, Anneliese Bukovac, Ramon O. Tasan,

Günther Sperk^*^

Department of Pharmacology, Medical University Innsbruck, 6020 Innsbruck, Austria

Methods

Table S1. Sequences of oligonucleotide probes

| **Probe** | **Gene bank No.** | **Bases** | **DNA Sequence** | **used** |
| --- | --- | --- | --- | --- |
| HDAC4 | NM_207225.1 | 384-414 | ACA ACT GCT CAT GTT GAC GCT GGA ACT CTG C | x |
| HDAC4 | NM_207225.1 | 2096-2126 | GTT GGT GTT CCC ACA GGT GCA CTG ATG CTT C |  |
|  |  |  |  |  |
| HDAC5 | NM_001077696.1 | 2990-3026 | ATG CAG GGA GAT GTA GAG CAC AGA GGG ATC GTT GTA G | x |
|  |  |  |  |  |
| HDAC6 | NM_010413.3 | 775-811 | GGG TTG TCT CCA TCA GAT CAA TGT ATT CCA GGC TGT G | x |
| HDAC6 | NM_010413.3 | 860-897 | GCA GGC ACA GGA ATA TGA GTT CGG ATG CAG ATA CAC TG |  |
|  |  |  |  |  |
| HDAC7 | NM_001204281 | 413-447 | GCT ACG GCA CTT CGC TTG CTC TTG TCT TTA TTG AG | x |
|  |  |  |  |  |
| HDAC9 | NM_024124.3 | 319-351 | GAG CTG ATC ATA CTG TGC ATT CTT TGC TGA GCC | x |
| HDAC9 | NM_024124.3 | 536-569 | GAT GCT GCC TTG TCA AAT TCT CGT GCT GCT TCT G |  |
|  |  |  |  |  |
| HDAC9,  var. 1 | NM_001271386.1 | 74-103 | TCA ACC TCT GAG CTA CAT CCA TAG TCC AGC | x |
| HDAC9,  var. 2 | NM_024124.3 | 2843-2873 | TGG ACC ACC ATG CTC TAC ATT CCT TCT CAG C | x |
|  |  |  |  |  |
| HDAC10 | NM_199198.2 | 402-432 | CAA CTC TTC CTC TGA TGC CTC ACA AGC TGA C | x |
| β-actin | NM_007393.5 | 971-1011 | CCA GAC AGC ACT GTG TTG GCA TAG AGG TCT TTA CGG ATG TC | x |
| NSE | 1102-1134 | NM_01350 | GTC TTC AAT GGA GAC CAC AGG ATA GTT CCG GAC | x |

Results

**Supporting Figure 1:**

**
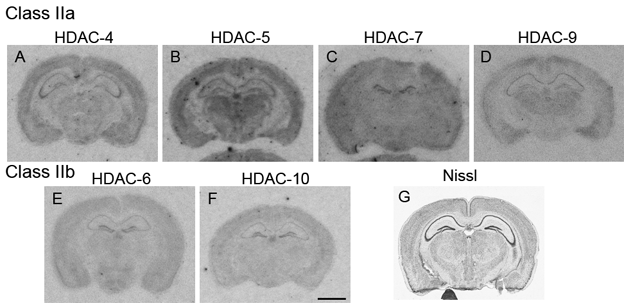
**

**Fig. S1. Expression patterns of mRNAs encoding class II HDACs in control brains.** The images represent photomicrographs of autoradiographs after *in situ* hybridization with the respective radiolabelled antisense probes. Panel G shows a photomicrograph of a Nissl stained section. Scale bar in F (for A – F): 2 mm.

**Supporting Figure 2:**

**
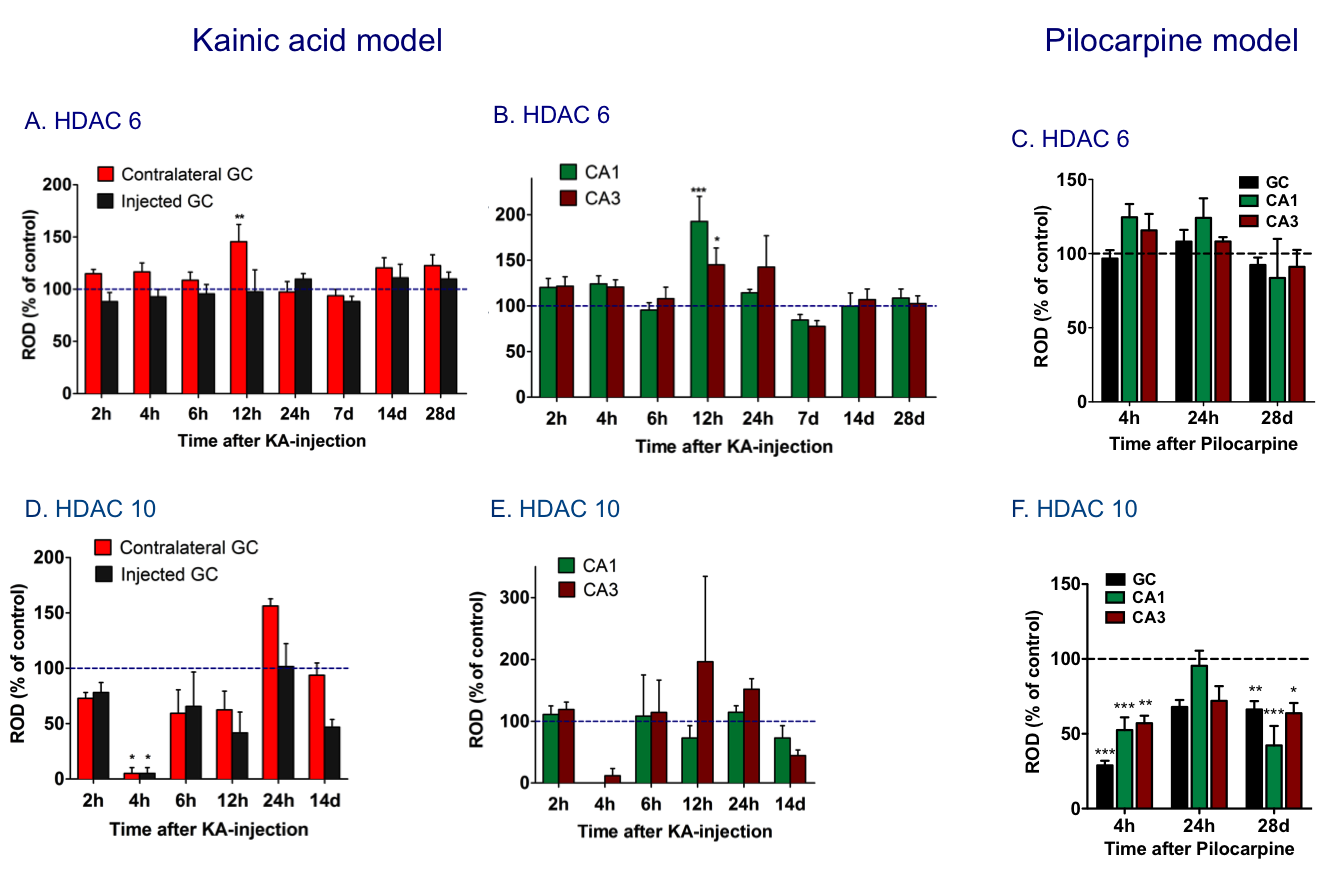
**

**Fig. S2. Time course of changes in expression of class IIb HDAC (HDAC 6 and 10) mRNAs in the kainate and pilocarpine model.**

The figures show changes in the mRNA expression as relative optic densities expressed as % of controls ± SEM. The left column (panels A, D) depicts changes in mRNA expression in the granule cell layer of the dentate gyrus (GC) in the KA model comparing the injected (black bars) with the contralateral side (red bars). The middle column (panels B, E) shows changes in the pyramidal cell layers (green bars, CA1; brown bars, CA3) contralateral to the injection side, which is not affected by cell loss upon local injection of KA. The right column (panels C, F) depicts changes in the pilocarpine model (black bars, granule cell layer; green bars and brown bars, CA1and CA3 pyramidal cell layers, respectively). Note the decreases in HDAC10 expression in both models during the acute status epilepticus. The numbers of animals used are given in the legend to Fig. 4. Statistical analysis was done by one-way ANOVA with Dunnett’s post-hoc test. * p<0.05, ** p<0.01, *** p<0.001 vs. controls.

**Supporting Figure 3:**


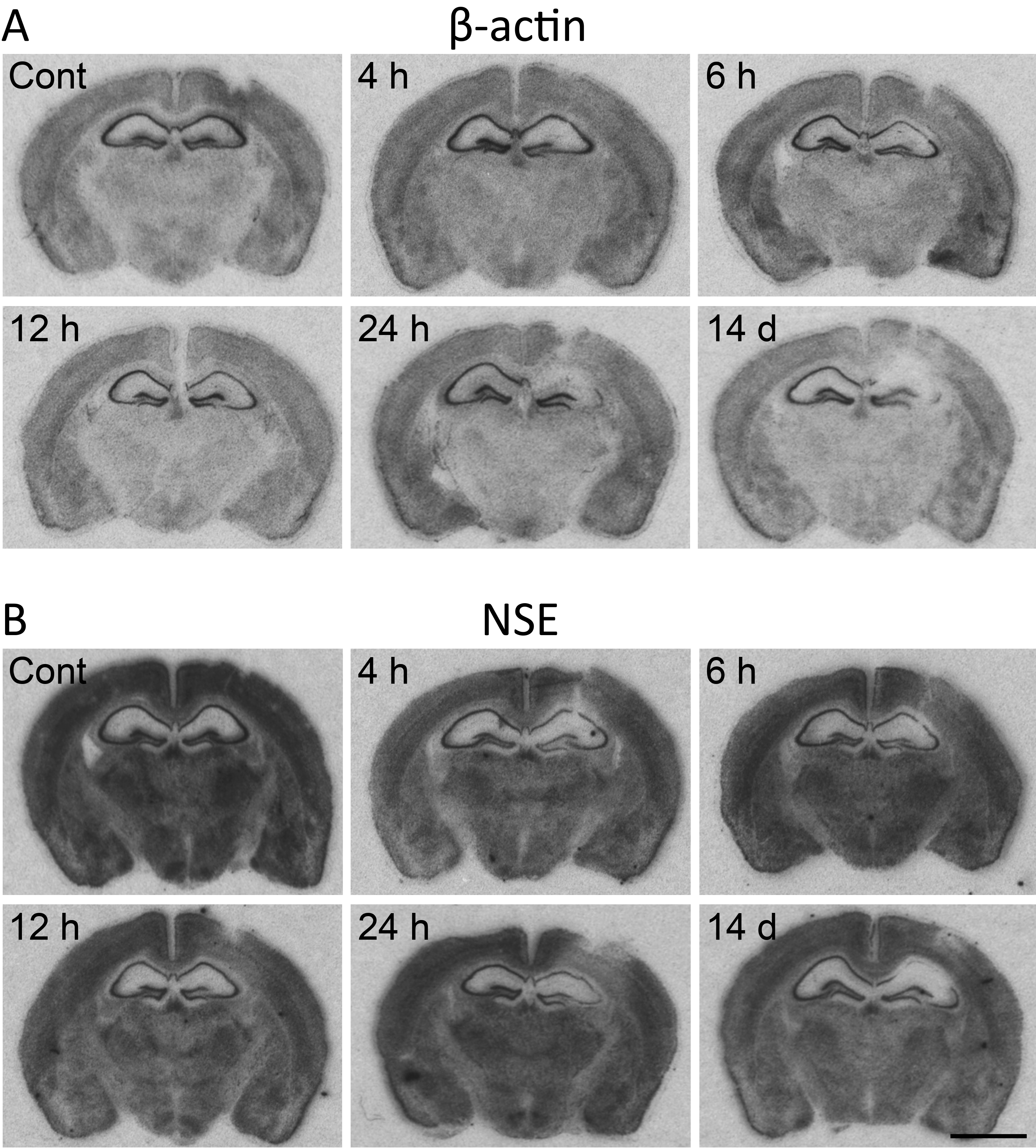


**Fig. S3.**

*In situ* hybridization for β-actin (A) and neuron specific enolase (NSE, B) mRNAs in a controls (Cont) and 4 h, 6 h ,12 h, 24 h and 14 d after intra-hippocampal KA injection (right hemisphere). Scale bar in B = 2 mm.

**Supporting Tables 2 and 3.**

**Table S2.** Densities of hippocampal cell layers after local intra-hippocampal injection of KA. Nissl stained sections (see Fig. 2) were scanned and gray values of individual hippocampal cell layers were determined using the ImageJ program and converted to relative optic densities (ROD). The data are expressed as % of controls ± SEM. Statistics: ANOVA with Dunnett’s post-hoc test. *p<0.05, **p<0.01, ***p<0.001.

|  |  | Dentate gyrus | | CA1 | | CA3 | | |
| --- | --- | --- | --- | --- | --- | --- | --- | --- |
|  |  | injected | contralateral | injected | contralateral | injected | contralateral | |
|  | n | ROD (% ± SEM) | | | | | | |
| Control | 6 | 100 ± 4.41 | | 100 ± 4.01 | | 100 ± 3.43 | | |
| KA, 4 h | 4 | 92.1 ± 4.43 | 89.0 ± 1.49 | 17.7 ± 19.05*** | 89.8 ± 6.35 | 38.6 ± 4.16*** | | 93.6 ± 2.92 |
| KA, 6 h | 6 | 89.7 ± 6.46 | 101.6 ± 9.20 | 22.1 ± 14.92*** | 95.5 ± 11.60 | 37.5 ±19.41*** | | 96.3 ± 9.69 |
| KA, 14 d | 6 | 69.8 ± 10.0** | 118.6 ± 4.14** | 28.0 ± 4.72*** | 105.3 ± 7.72 | 79.6 ± 17.81* | | 107.8 ± 6.12 |
| KA, 28 d | 4 | 72.9 ± 18.0** | 97.9 ± 5.19 | 42.2 ± 23.63*** | 109 ± 12.79 | 71.4 ± 13.27** | | 104.9 ±12.38 |

**Table S3.** Density of hippocampal cell layers after pilocarpine-induced status epilepticus. Nissl stained sections (see Fig. 2) were scanned and gray values of individual hippocampal cell layers were determined using the ImageJ program and converted to relative optic densities (ROD). The data are expressed as % of controls ± SEM. Statistics: ANOVA with Dunnett’s post-hoc test. *p<0.05, **p<0.01, ***p<0.001.

|  |  | Dent. gyrus | CA1 | CA3 |
| --- | --- | --- | --- | --- |
|  | n | ROD (% ± SEM) | | |
| Control | 6 | 100 ± 4.01 | 100 ± 2.24 | 100 ± 3.45 |
| Pilo, 4 h | 6 | 97.7 ± 2.79 | 63.6 ± 4.67*** | 94.2 ± 2.82* |
| Pilo, 24 h | 5 | 101.4 ± 2.82 | 78.15± 7.01*** | 98.2 ± 2.19 |
| Pilo, 28 d | 4 | 104.4 ± 2.41 | 73.0± 5.75*** | 92.6 ± 4.02** |
